# Factors associated with spontaneous preterm birth in Addis Ababa public hospitals, Ethiopia: cross sectional study

**DOI:** 10.1186/s12884-018-1957-0

**Published:** 2018-08-13

**Authors:** Ababe Tamirat Deressa, Amsale Cherie, Teshome Melese Belihu, Gemechu Ganfure Tasisa

**Affiliations:** 1 School of Nursing and Midwifery, College of medicine and health sciences, Hawassa University, Hawassa, Ethiopia; 2 Department of Nursing and midwifery, School of Allied health sciences, Addis Ababa University, Addis Ababa, Ethiopia; 3 School of health Sciences, College of medicine and health sciences, Hawassa University, Hawassa, Ethiopia; 4 Midwifery department, School of health sciences, MaddaWalabu University, Bale Robe, Ethiopia

**Keywords:** Preterm birth, Spontaneous, Factors, Addis Ababa

## Abstract

**Background:**

Spontaneous preterm birth is commencement of labor with intact or pre labor rapture of membrane and birth before 37 weeks of gestation. The aim of this study was to identify common factors associated with spontaneous preterm birth in Addis Ababa public hospitals.

**Methods:**

After random selection of three hospitals from the six Addis Ababa’s Public hospitals having Neonatal intensive care unit, systematic sampling was employed to select study units from admission log book of the neonates. Data were collected using structured checklist. Finally, data entered to EpiData 3.1 and transported to SPSS 22 for analysis. Bivariate and multivariate logistic regression analysis was done for the variables.

**Result:**

The mean gestational age of preterm birth was 32.45 (±2.903 SD). Majority (66.1%) of preterm births were spontaneous and 33.9% were induced preterm births. Hypertension during pregnancy [*P* = 0.001, AOR = 0.182, 95% CI: (0.067, 0.493)] and maternal HIV infection [*P* = 0.041**,** AOR **=** 3.408 95% CI: (1.048, 11.079)] significantly associated with spontaneous preterm birth.

**Conclusion:**

Those mothers who were diagnosed with hypertension during pregnancy less likely gave spontaneous preterm birth than who had no history of hypertension during pregnancy and HIV positive mothers gave spontaneous preterm more likely than HIV negative mothers. Thus, giving emphasis to these factors with appropriate care during pregnancy is important to reduce spontaneous preterm birth.

## Background

Preterm birth is defined as a delivery which occurs at less than 37 completed weeks of gestation. It is classified as extremely preterm (< 28 weeks), very preterm (28 to < 32 weeks), and moderate to late preterm (32 to < 37 weeks). Likewise preterm birth can be categorized on the basis of birth weight. Neonates weighting less than 2500 g are classified as low birth weight (LBW), < 1500 g are very low birth weight (VLBW) and < 1000 g are extremely low birth weight (ELBW). Preterm birth can also be spontaneous or provider initiated (induced). Spontaneous preterm birth is commencement of labor with intact or pre labor rapture of membrane and birth before 37 weeks of gestation. Spontaneous onset of labor accounts for 65–70% of all preterm births and provider initiated for 30–35% [1].

The rate of preterm birth is escalating globally and ranges from 5 to 7% in developed countries and significantly higher in least developed countries. Of the global 135 million live births, 14.9 million (11.1%) babies were born preterm in 2010. More than 60% of preterm births occurred in Sub-Saharan Africa and 9.1 million (12.8%) in south Asia [2].

The rate of preterm birth in Southeast Nigeria rose from 9.8% in 2009 to 17.1% in 2013 after peaking at 23% in 2012. Approximately 57% of preterm births were spontaneous preterm births while provider-initiated births occurred in 43% of them. In Gondar University hospital, Northwest Ethiopia about one in seven adverse birth outcomes (14.3%) was found to be preterm birth and in Mettu Karl hospital, Southwestern Ethiopia, the recorded rate of preterm delivery in mothers having pregnancy related hypertension was 31.4% [3–5].

Preterm births have different causes and risks of mortality, morbidity, impaired growth, and non-communicable diseases. Thus, being born preterm predisposes infants to higher risks of chronic diseases and mortality later in life. Specially, infants born before 32 weeks of gestation are at high risk of adverse health outcomes [6–12]. This study aimed to contribute in identifying factors associated with spontaneous preterm birth in Addis Ababa Public hospitals having Neonatal Intensive Care Unit from 2011 to 2015.

## Methods

Cross sectional study design was applied for this study. From the six public hospitals having neonatal intensive care unit in Addis Ababa, three of them were selected with simple random sampling by lottery method. The sample was proportionally allocated to each three hospitals based on their number of preterm admissions to their respective neonatal intensive care unit. The medical records of the preterm neonates were selected from the registry log book of the neonatal intensive care unit using systematic sampling. The medical record of the preterm neonate admitted from referral within the three selected hospital was excluded to reduce chance of repetition. Retrospective data were collected from the five years admissions of preterm births to neonatal intensive care unit. Data was collected by using structured, pretested checklist from the selected patient’s medical records. The checklist was adopted and modified from different related studies [2, 5, 13–21].

Data coding and entry was accomplished using EpiData 3.1 and exported to SPSS version 22. Data cleaning, recoding and analysis were performed with this SPSS. Bivariate logistic regression analysis was done after dichotomizing the dependent variables with coding ‘1’ for being spontaneous preterm birth and ‘0’ for not being spontaneous preterm birth. Multivariate logistic regression analysis was performed to control confounding factors in the association. *P*-value of < 0.05 was used to express statistical significance of the variables. Sentence, table of frequency and graphs were used present result of the study.

## Results

From total 23,115 admissions to NICU, preterm accounts 3732 giving Prevalence of preterm birth from neonatal intensive care unit admission 16.15%. A total of 384 preterm neonates’ medical records having both maternal and the neonate’s histories were involved in this study and all data were used for analysis.

**Fig. 1:**
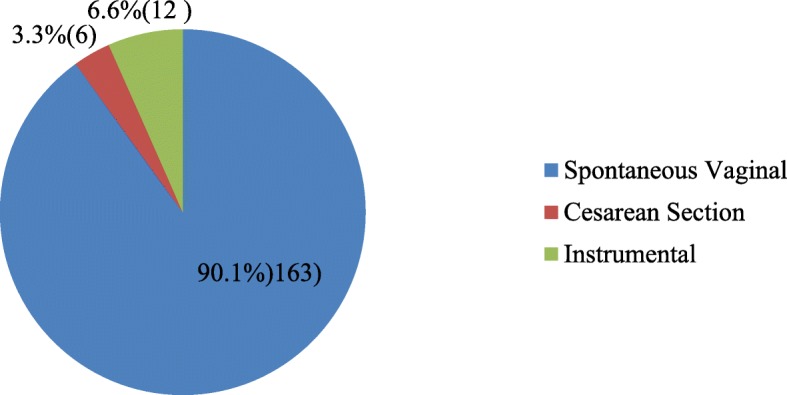
Immediate Previous mode of delivery, Addis Ababa, Ethiopia, April, 2016

The mean age of the mothers was 26.09 (± 4.772908 SD) which ranges of 14 to 43 and 26 was the median age. Majority of the mothers (51.7%) were above the mean age (≥ 26) where as 48.3% were younger than 25 years. One hundred ninety five (50.8%) and 203 (52.9%) were primigravida and primipara respectively. Spontaneous Vaginal delivery was the commonest (90.1%) immediate previous mode of delivery for those who have ever given births previously (Fig. 1). For the current (through which the preterm baby born) mode of delivery spontaneous vaginal accounts for 259 (67.4%), cesarean section 121 (32.5%) and instrumental delivery accounts about 4 (1%). In addition, 156 (40.6%) of the mothers have been diagnosed at least with one medical problem during the current pregnancy and 228 (59.4%) were not. From these, hypertension stands for 664 (2.3%), Premature rupture of membrane for 47 (30.1%), HIV for 24 (15.4%), ante-partum hemorrhage (APH) for 13 (8.3%), Chorioamnoitis for 12 (7.7%), diabetes for 4 (2.6%), and others for 7 (4%).

**Fig. 2:**
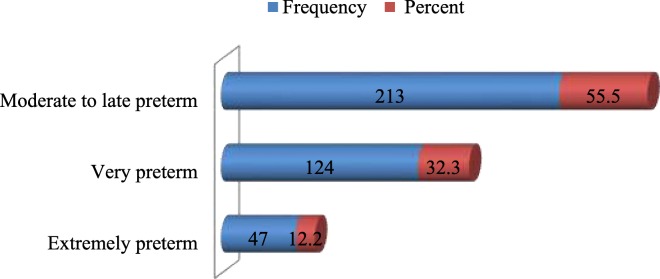
Maturity classification of preterm births, Addis Ababa, Ethiopia, April, 2016

One hundred ninety six (51%) of the preterm births were male and 188 (49%) were female neonates. Two hundred fifty four (66.1%) were spontaneous preterm births. The minimum and maximum gestational age at birth of preterm births were 24 and 36 weeks respectively with mean gestational age of 32.45 weeks (±2.903 SD) (Fig. 2). The first minute APGAR score of the preterm neonates was found to range 1 to 9 with mean of 5.64 and standard deviation of 1.592 where 261 (68%) and 123 (32%) were it the category of low (0–6) and normal (7–10) APGAR score respectively. According to finding of this study, 270 (70.3%) of preterm births’ weights were appropriate for their gestational age (AGA), 109 (28.4%) were small for their gestational age (SGA) and few [5 (1.3%)] were under category of large for gestational age (LGA).

In bivariate analysis; compared to mothers older than 26 years old, those younger than 25 years were 19.5% more likely to give spontaneous preterm birth (RR = 1.195, *P* = 0.015, OR = 1.702, 95% CI: 1.108,2.613). Spontaneous preterm births had an odds of being born to HIV mothers that are 3.828 times the odds of those induced (non-spontaneous) preterm birth (*P* = 0.008, OR = 3.828, 95% CI: 1.428,10.258). Mothers who experienced premature rupture of membrane more likely (*P* = 0.000, OR = 5.645, 95% CI: 2.589, 12.309) gave spontaneous preterm birth. Other variables like parity, gravidity, multiple pregnancies, outcome of immediate previous pregnancy, time and mode of previous delivery had not shown significant association with spontaneous preterm birth in this study.

In multivariate analysis, only hypertension during pregnancy [*P* = 0.001, AOR = 0.182, 95% CI: (0.067, 0.493)] and maternal HIV infection [*P* = 0.041**,** A0R **=** 3.408 95% CI: (1.048, 11.079)] remained associated with spontaneous preterm birth and other variables turned insignificant in multivariate analysis. Those mothers who were diagnosed with hypertension during pregnancy less likely gave spontaneous preterm birth than who had no history of hypertension during pregnancy and HIV positive mothers gave spontaneous preterm more likely than HIV negative mothers (Table 1).

**Table 1:** Multivariate Analysis of factors associated with Spontaneous Preterm birth, Addis Ababa, Ethiopia, April 2016

Characteristics	Spontaneous preterm		S.E.	*P*-value	AOR( 95% C.I)
	Yes: # (%)	No: # (%)			
Gravidity					
<1	135(69.2)	60(30.8)	1.579	0.686	0.528 (0.024, 11.659)
>2^a^	119(63.0)	70(37.0)			1
Parity					
<1	141(69.5)	62(30.5)	1.706	0.682	2.010 (0.071, 56.894)
>2^a^	113(62.4)	68(37.6)			1
Time of immediate previous delivery					
12 months ago	29(65.9)	15(34.1)	0.928	0.699	1.432 (0.232, 8.829)
36 months ago^a^	8(47.1)	9(52.9)			1
Multiple pregnancy					
Yes	44(60.3)	29(39.7)	0.545	0.904	1.068 (0.367, 3.109)
No^a^	210(67.5)	101(31.5)			1
Maternal HIV infection					
Yes	18(75.0)	6(25.0)	0.601	**0.041** ^**b**^	3.408 (1.048, 11.079)
No^a^	58(43.9)	74(56.1)			1
Hypertension during pregnancy					
Yes	13(19.7)	53(80.3)	0.509	**0.001** ^**b**^	0.182 (0.067, 0.493)
No^a^	63(70.0)	27(30.0)			1
PROM					
Yes	24(51.1)	23(48.9)	0.530	0.066	2.646 (0.937, 7.476)
No^a^	52(47.7)	57(52.3)			1
Age of mothers					
<25	135(72.2)	52(27.8)	0.696	0.919	0.932(0.238, 3.645)
>26^a^	119(60.4)	78(39.6)			

*S.E.* Standard error, *AOR* Adjusted Odds ratio

^a^Reference, ^b^Significant association

## Discussion

Prevalence of preterm birth in NICU admission in this study (16.5%) is higher than that of Gondar university hospital and lower than that of Mettu Karl hospital. The reason for this variation might be methodological differences employed in these previous studies [3, 5]. Majority of the preterm births (66.1%) were spontaneous and only few were induced deliveries. This finding is similar with global report and study in Southeast Nigeria in which spontaneous preterm accounts greater proportion [4, 22]. Majority of the preterm birth’s mothers (51.7%) in this study were above the mean age (≥ 26 years), but in rural South Africa and Malawi, the greater proportion of the mothers were younger than 20 years. Methodological and socio-economic variation can be the reason for this difference. Unlike study in Nova Scotia which reported older age to be at higher risk to give spontaneous preterm birth, mothers who were younger than 25 years in this study were at higher risk to give spontaneous preterm birth in bivariate analysis (RR = 1.195, *P* = 0.015, OR = 1.702, 95% CI: 1.108,2.613). Socioeconomic difference with methodology of the study can be reason into account for this variation. But finding in this study is in line with evidence of study from Taiwan for risk of preterm birth. Even though age classification for the association varies, study in Malawi also supports this as younger women are at high risk of giving preterm birth [23, 24]. As global report indicated [22], premature rupture of membrane was associated with spontaneous preterm birth. Also this study verified that mothers who developed premature rupture of membrane had greater odds of giving spontaneous preterm birth which is 5.645times odds of those who haven’t developed premature rupture of membrane. But it is left on the border of significance in multivariate analysis. Although report of preterm delivery for immediate previous pregnancy was high (24.9%) like that of report from Malawi, statistical significance was not observed in this study [15]. Mothers with HIV had greater odds of giving spontaneous preterm birth which shown significant statistical association in both bivariate and multi-variate analysis (*P* = 0.041, AOR = 3.408, CI: 1.048, 11.079). Similarly, HIV disease significantly associated with increased risk of giving preterm birth in Tanzania [25].

## Conclusion

Age of mothers younger than 25 years, having at least one medical disorder during pregnancy, maternal HIV infection, hypertension during pregnancy, premature rupture of membrane from which maternal HIV infection and hypertension during pregnancy remained significant in multivariate analysis were factors found to have significant association with spontaneous preterm birth.

## Abbreviations

AGA: Appropriate for gestational age
AOR: Adjusted Odd ratio
CI: Confidence Interval
ELBW: Extremely low birth weight
HIV: Human Immune-deficiency Virus
LBW: Low birth weight
LGA: Large for gestational age
OR: Odds Ratio
RR: Relative Risk
SD: Standard Deviation
SGA: Small for gestational age
VLBW: Very low birth weight
